# The link between serum cotinine levels and gallstones prevalence in adults: a cross-sectional analysis using NHANES data (2017–2020)

**DOI:** 10.3389/fnut.2024.1438170

**Published:** 2024-09-10

**Authors:** Xin Liu, Zheng Zhang, Haoran Wang, Shah Faisal, Meng He, Sheng Tai, Yujia Lin

**Affiliations:** ^1^Department of Hepatobiliary Surgery, Second Affiliated Hospital of Harbin Medical University, Harbin, Heilongjiang, China; ^2^Department of Hepatobiliary Surgery, First Affiliated Hospital of Harbin Medical University, Harbin, Heilongjiang, China

**Keywords:** gallstones, serum cotinine, tobacco exposure, smoking, NHANES

## Abstract

**Background:**

Gallstones represent a prevalent health issue globally, resulting in significant annual healthcare costs. While tobacco exposure is recognized for its association with numerous diseases, its correlation with gallstones remains contentious. Serum cotinine, a metabolite of nicotine, serves as a widely utilized indicator for assessing tobacco exposure. Crucially, no research has yet examined the association between serum cotinine levels and the gallstones.

**Methods:**

This study is designed as a cross-sectional analysis, utilizing data from the NHANES public database. The relationship between serum cotinine levels and gallstones was analyzed using multinomial logistic regression models and smooth curve fitting. Subgroup analyses and interaction tests were performed to examine the potential contributions of different populations and covariates to the findings.

**Results:**

A total of 5,856 participants were included in this study. After adjusting for relevant covariates, the multiple logistic regression model results indicated that for each unit increase in serum cotinine concentration above 0.29 ng/mL, there was a 29% increase in the prevalence of gallstones. Furthermore, smooth curve fitting analysis revealed a positive correlation between these variables. These findings underscore the impact of tobacco exposure on gallstone prevalence.

**Conclusion:**

This study demonstrates a positive correlation between tobacco exposure, as measured by serum cotinine levels, and the prevalence of gallstones, thus adding to the body of existing research on this relationship.

## 1 Introduction

Gallstones are one of the most prevalent hepatobiliary disorders, affecting 10 to 15% of adults in Western countries and placing a considerable burden on healthcare systems. Despite their prevalence, the etiology and pathogenesis of gallstones are not fully understood, and there is a notable lack of effective non-surgical treatment options (1, 2). Previous studies have identified several primary risk factors for gallstones, including genetic predisposition, elevated cholesterol secretion, and bile supersaturation. Additional risk factors encompass obesity, female gender, advancing age, type 2 diabetes, and physical inactivity (3, 4). Gallstones commonly originate within the wall of the gallbladder or the biliary tract and can manifest either as asymptomatic or symptomatic conditions (4). They are categorized based on their primary components into pure cholesterol stones, pure pigment stones, or mixed stones (5, 6), pure cholesterol stones predominantly comprise 90% cholesterol, whereas bile pigment stones, containing 90% bilirubin, are further distinguished as either brown or black pigment stones (7, 8). Gallstones predominantly consist of either cholesterol or melanin, with brown pigment stones being the most common type among bile duct stones (9). Moreover, besides the pain associated with gallstone disease itself, complications such as biliary pancreatitis can arise, potentially leading to life-threatening situations. Worldwide, gallstones stand as the primary etiology of acute pancreatitis, with an augmented risk correlated to the escalating quantity and dwindling size of stones (10, 11).

Cotinine, a major metabolite of nicotine, serves as a widely utilized biomarker for assessing population tobacco exposure, with its concentration in blood, urine, and saliva commonly measured (12, 13). Studies have demonstrated that serum cotinine concentrations offer a more reliable indicator of tobacco exposure compared to self-reported smoking numbers (14, 15). Furthermore, serum cotinine concentration is also employed as an effective measure of smoking status (16).

Tobacco exposure has emerged as the most significant preventable public health issue globally, with various toxic substances produced by tobacco combustion contributing to a multitude of diseases and fatalities (17). However, the association between tobacco exposure and gallstones remains contentious (18), while some studies have reported a heightened prevalence of gallstones among smokers (19–21), others contend that no such relationship exists (22–25) Therefore, this study aimed to delve deeper into the correlation between tobacco exposure and gallstones, utilizing serum cotinine concentration as an indicator of tobacco exposure.

## 2 Methods and materials

### 2.1 Study population and design

NHANES is a large cross-sectional survey designed to represent the health and nutrition conditions of the US population, with each cycle spanning two years. The survey includes demographic, socioeconomic, nutritional, and health-related questions in interviews. Approval for NHANES research protocols was obtained from the National Center for Health Statistics (NCHS) Research Ethics Review Board, and written informed consent was provided by all participants.

**Figure 1:**
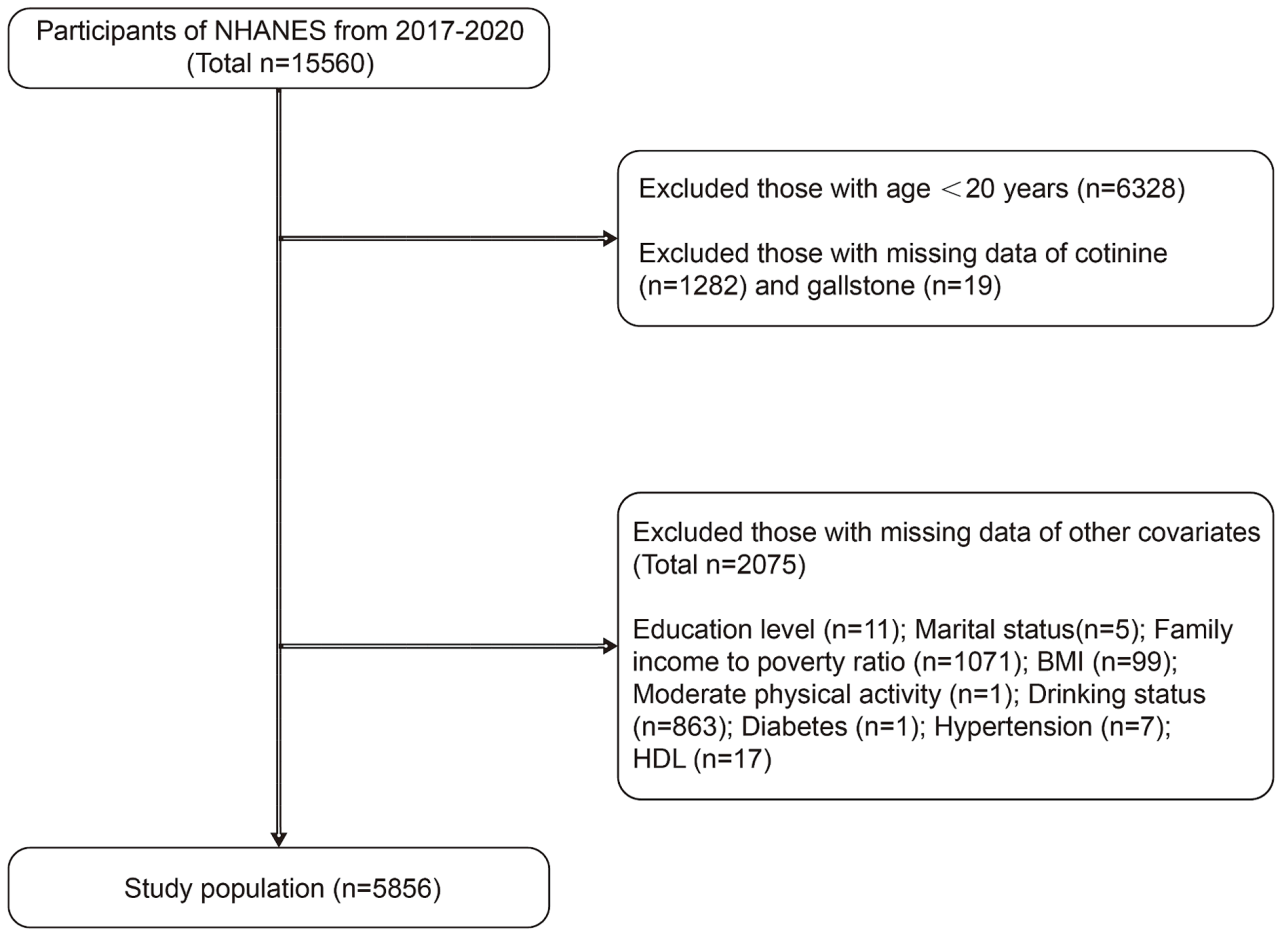
After inclusion and exclusion criteria screening, a total of 5,856 subjects were included in this study.

This study utilized data from the NHANES database spanning the years 2017 to 2020, encompassing a survey population of 15,560 participants. Initially, exclusion criteria were applied, leading to the removal of 6,328 participants under the age of 20. Subsequently, 1,282 participants with missing data on the exposure variable (serum cotinine), 19 participants with missing outcome data (gallstones), and finally, 2075 participants with missing covariates were excluded from the analysis. This resulted in a final analysis sample of 5,856 participants. The detailed process of inclusion and exclusion criteria is illustrated in Figure 1.

### 2.2 Definition of gallstones

A specific questionnaire within the NHANES database contained the inquiry: “Has a doctor or other health professional ever diagnosed you with gallstones?” Respondents answering affirmatively were classified as having gallstones, while those responding negatively were categorized as gallstone-free. Subjects who declined to respond or answered “do not know” were omitted from the study.

### 2.3 Serum cotinine

Serum cotinine, serving as the exposure variable in this study, was quantified using an isotope-dilution high-performance liquid chromatography/atmospheric pressure chemical ionization tandem mass spectrometry (ID HPLC-APCI MS/MS) method. We stratified serum cotinine concentrations into tertiles to investigate the dose–response association between exposure and outcome.

### 2.4 Covariates

All covariates for this study were derived from demographic, examination, laboratory, and questionnaire data obtained from the NHANES database. Confounding factors included age, gender, race, education level, marital status, ratio of family income to poverty (PIR), body mass index (BMI), physical activity, drinking status, diabetes, hypertension, direct HDL-cholesterol (HDL), and total cholesterol (TC). Age was categorized into groups based on 40 and 60 years old, while race and marital status were classified according to NHANES classifications. Education level was divided into three groups according to completion of high school education. PIR was categorized using cut-off points of 1.3 and 3.5 as previously defined (26), BMI was used to differentiate between overweight and obesity, with thresholds set at 25 kg/m^2^ and 30 kg/m^2^, respectively. Physical activity levels were determined based on responses to questions about engaging in vigorous-intensity and moderate-intensity activities for at least 10 min continuously. Positive responses to both were categorized as engaging in vigorous or moderate activity. Regarding drinking status, participants were asked, “How often have you consumed alcoholic beverages in the past 12 months?” Those reporting consumption less than once a month were categorized as non-drinkers. Diabetes and hypertension status were determined based on responses to questions regarding prior diagnoses by a doctor or health professional. Data on HDL and TC were obtained from NHANES laboratory records. The selection of covariates in this study was guided by three key principles: identifying relevant covariates from previous studies utilizing the NHANES database for gallstone research (27–29), incorporating known risk factors from comprehensive review on gallstones (18), and ensuring that the selected covariates were available within the NHANES database for further analysis. This approach ensured that the covariates included in the analysis were both relevant and supported by existing literature and available data.

### 2.5 Statistical analysis

Continuous variables were reported as means ± standard errors (SEs), while categorical variables were presented as numbers and percentages. The Kruskal-Wallis Rank Test was utilized for continuous variables, and Fisher’s Exact Test was applied for categorical variables, particularly when the expected count was less than 10. To explore the relationship between serum cotinine and gallstones across different models, multinomial logistic regression models were employed. Three statistical models were established: the Non-adjusted model, with no covariate adjustments; Adjusted Model I, which included adjustments for age, gender, and race; and Adjusted Model II, incorporating adjustments for age, gender, race, education level, marital status, ratio of family income to poverty (PIR), body mass index (BMI), physical activity, drinking status, diabetes, hypertension, high-density lipoprotein (HDL), and total cholesterol (TC). Additionally, smooth curve fitting (penalized saline method) was conducted to further examine the association between gallstones and serum cotinine. Subsequently, subgroup analyses of all covariates were performed to investigate potential modifications in the association between exposure and outcome. These covariates were also considered as interaction terms, and interaction tests were conducted. In the interaction tests and subgroup analyses, HDL and TC were additionally converted from continuous variables to tertile categorical variables. All statistical analysis processes were carried out based on EmpowerStats^1^ and statistical significance was defined as a *p* value below 0.05.

## 3 Results

### 3.1 Baseline characteristics of participants

After establishing reasonable inclusion and exclusion criteria, a total of 5,856 participants aged 20 years and above were included in this study. In Table 1, there were 5,229 subjects without gallstones and 627 subjects with gallstones. We found significant differences (*p* < 0.05) between the two groups of participants with and without gallstones in terms of age, gender, race, marital status, BMI, physical activity level, drinking status, diabetes, hypertension, and serum cotinine grouping.

**Table 1:** Baseline characteristics of participants (*N* = 5,856).

Characteristic	Non-stone formers (*n* = 5,229)	Stone formers (*n* = 627)	*p*-value
Age (years)			<0.001
20–39	1,689 (32.30%)	101 (16.11%)	
40–59	1755 (33.56%)	211 (33.65%)	
≥ 60	1785 (34.14%)	315 (50.24%)	
Gender, *n* (%)			<0.001
Male	2,774 (53.05%)	181 (28.87%)	
Female	2,455 (46.95%)	446 (71.13%)	
Race, *n* (%)			<0.001
Mexican American	606 (11.59%)	82 (13.08%)	
Other Hispanic	501 (9.58%)	71 (11.32%)	
Non-Hispanic White	2000 (38.25%)	293 (46.73%)	
Non-Hispanic Black	1,357 (25.95%)	112 (17.86%)	
Other Race	765 (14.63%)	69 (11.00%)	
Education level, *n* (%)			0.425
Below high school	808 (15.45%)	105 (16.75%)	
High school	1,262 (24.13%)	160 (25.52%)	
Above high school	3,159 (60.41%)	362 (57.74%)	
Marital Status, *n* (%)			<0.001
Cohabitation	3,053 (58.39%)	386 (61.56%)	
Solitude	1,156 (22.11%)	170 (27.11%)	
Never married	1,020 (19.51%)	71 (11.32%)	
PIR, *n* (%)			0.056
<1.3	1,399 (26.75%)	161 (25.68%)	
≥1.3–<3.5	2043 (39.07%)	275 (43.86%)	
≥ 3.5	1787 (34.17%)	191 (30.46%)	
BMI (kg/m^2^), *n* (%)			<0.001
<25	1,352 (25.86%)	66 (10.53%)	
≥25–<30	1,660 (31.75%)	172 (27.43%)	
≥30	2,217 (42.40%)	389 (62.04%)	
Physical activity, *n* (%)			
Vigorous			<0.001
Yes	1,363 (26.07%)	97 (15.47%)	
No	3,866 (73.93%)	530 (84.53%)	
Moderate			0.002
Yes	2,218 (42.42%)	226 (36.04%)	
No	3,011 (57.58%)	401 (63.96%)	
Drinking status, *n* (%)			<0.001
Yes	2,445 (46.76%)	382 (60.93%)	
No	2,784 (53.24%)	245 (39.07%)	
Diabetes, *n* (%)			<0.001
Yes	725 (13.86%)	163 (26.00%)	
No	4,504 (86.14%)	464 (74.00%)	
Hypertension, *n* (%)			<0.001
Yes	1917 (36.66%)	332 (52.95%)	
No	3,312 (63.34%)	295 (47.05%)	
HDL (mg/dL)	53.71 ± 16.23	53.07 ± 15.23	0.541
TC (mg/dL)	185.96 ± 41.23	184.99 ± 43.51	0.469
Serum cotinine (ng/mL)			0.030
T1 (0–0.01)	1706 (32.63%)	236 (37.64%)	
T2 (0.01–0.29)	1758 (33.62%)	204 (32.54%)	
T3 (0.29–1620.00)	1765 (33.75%)	187 (29.82%)	

Continuous variables are described as means ± SEs. Categorical variables are presented as numbers (percentages). *p* value: Kruskal Wallis Rank Test for continuous variables, Fisher Exact for categorical variables with Expects < 10. PIR, Ratio of family income to poverty; BMI, Body Mass Index; HDL, Body Mass Index; TC, Total Cholesterol.

Among the participants with gallstones specifically, individuals aged 60 years and above accounted for a higher proportion (50.24%), while females had a higher percentage (71.13%) compared to males (28.27%). Regarding marital status distribution among those with gallstones versus those without them, cohabitation and solitude had a higher proportion among patients with gallstones than those without them. In terms of BMI distribution, overweight participants accounted for as much as 62.04% of patients with gallstones. Concerning physical activity levels, non-exercisers constituted a larger proportion of patients with gallstones compared to those without them. The proportions regarding drinking status showed an inversion between participants with and without stones, as drinkers represented approximately 60.93% of patients with gallstones. Participants diagnosed with diabetes or hypertension had higher proportions within the group of patients suffering from gallstones compared to those who did not have them.

### 3.2 Relationship between serum cotinine and gallstones

**Figure 2:**
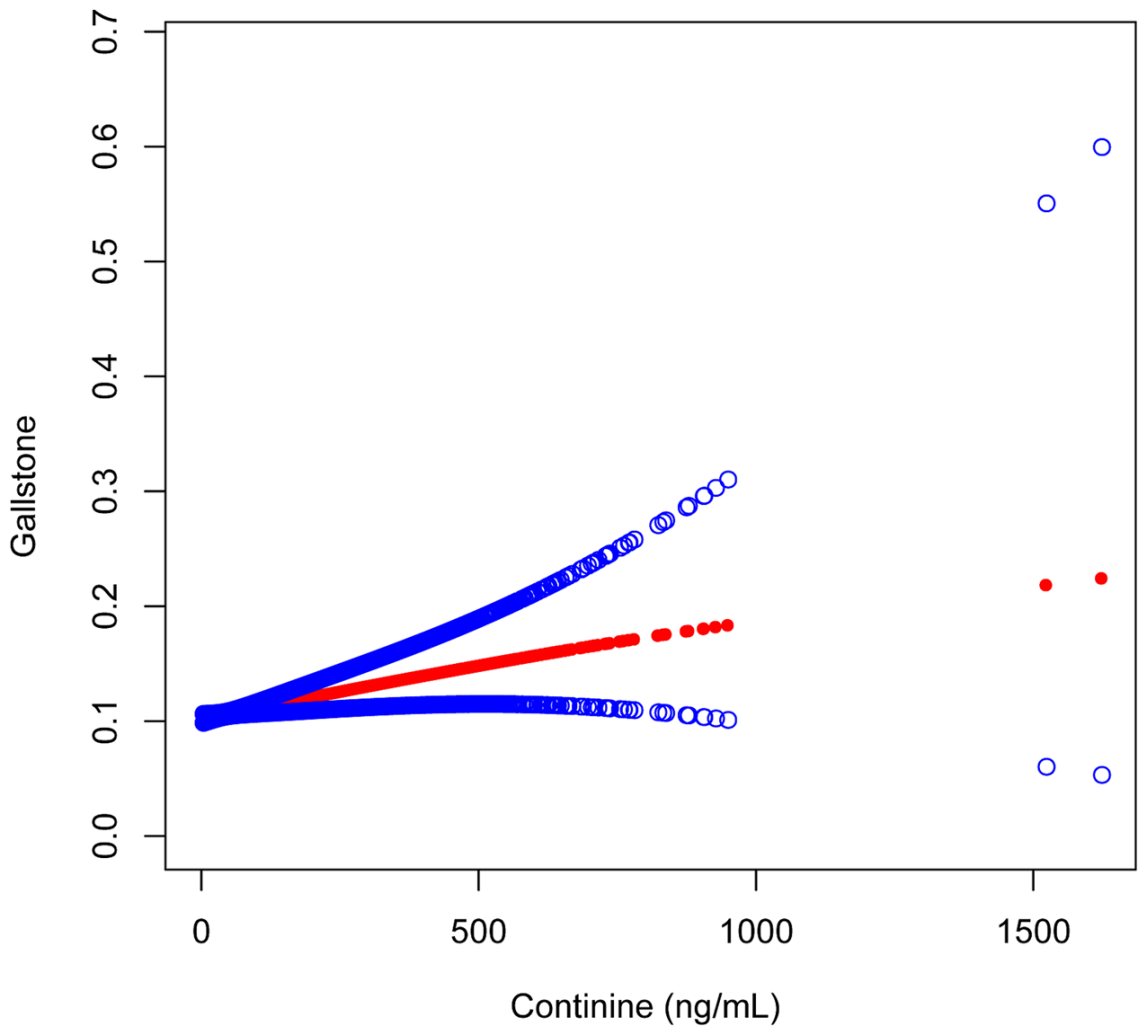
Association between serum cotinine and gallstones.

The serum cotinine concentration exhibited a positive correlation with the prevalence of gallstones. In the Adjust II model presented in Table 2, an association was observed between serum cotinine levels within the T3 range (0.29–1620.00 ng/mL) and a 29% increase in gallstone prevalence (OR = 1.29; 95% CI: 1.01–1.63; *p* = 0.0374). Furthermore, as the concentration of cotinine increased, this positive correlation demonstrated a gradual increment (*p* for trend = 0.0149). Smooth curve fitting analysis further substantiated the existence of a positive correlation between these two variables (Figure 2).

**Table 2:** Relationship between serum cotinine and gallstone in different models.

Exposure	Non-adjusted OR (95% CI) (*n* = 5,856)	Adjust I OR (95% CI) (*n* = 5,856)	Adjust II OR (95% CI) (*n* = 5,856)
Serum cotinine (ng/mL)			
T1 (0–0.01)	1.0	1.0	1.0
T2 (0.01–0.29)	0.84 (0.69,1.02) *p* = 0.0833	1.04 (0.85,1.28) *p* = 0.6869	0.99 (0.80,1.23) *p* = 0.9421
T3 (0.29–1620.00)	0.77 (0.63,0.94) *p* = 0.0100	1.24 (1.00,1.55) *p* = 0.0537	1.29 (1.01,1.63) *p* = 0.0374
*P* for trend	0.0488	0.0483	0.0149

Non-adjusted model adjusts for: None. Adjust I model adjusts for age, gender, race. Adjust II model adjusts for age, gender, race, education level; marital status; PIR; BMI; physical activity; drinking status; diabetes; hypertension; HDL and TC. PIR; Ratio of family income to poverty; BMI, Body Mass Index; HDL, Body Mass Index; TC, Total Cholesterol.

### 3.3 Subgroup analysis and interaction tests for the associations between serum cotinine and gallstones

In the subgroup analysis, all covariates were also treated as interaction terms and underwent interaction testing. These covariates were identical to those used in the final adjusted model (Adjusted II model), with one necessary exception: during the subgroup analysis for each specific covariate, that particular covariate was not included as an adjusted variable in its own analysis. Additionally, HDL and TC were converted from continuous variables to tertile categorical variables. In each analysis, all covariates except those under examination were adjusted. The findings indicated that none of the subgroups exhibited a significant impact on the relationship between serum cotinine concentration and gallstones (Table 3).

**Table 3:** Subgroups analysis and interaction tests for the associations between serum cotinine and gallstones.

Subgroup	Serum cotinine (ng/mL) OR and 95% CI			*p* for Interaction
	T1 (0–0.01)	T2 (0.01–0.29)	T3 (0.29–1620.00)	
Age (years)				0.1385
20–39	Ref.	1.42 (0.78, 2.56)	1.71 (0.92, 3.18)	
40–59	10.81 (1.05, 110.81)	10.87 (1.06, 111.37)	18.16 (1.78,184.99)	
≥ 60	54.97 (6.00,504.07)	50.09 (5.45, 460.09)	51.21 (5.55,472.33)	
Gender				0.1951
Male	Ref.	0.84 (0.57, 1.24)	0.96 (0.63, 1.44)	
Female	4.76 (0.94, 24.02)	5.22 (1.04, 26.27)	7.21 (1.43, 36.32)	
Race				0.9793
Mexican American	Ref.	1.24 (0.68, 2.25)	1.75 (0.80, 3.82)	
Other Hispanic	11.45 (0.54, 241.09)	11.29 (0.55, 233.21)	14.32 (0.67,306.35)	
Non-Hispanic White	8.69 (0.89, 84.78)	7.75 (0.80, 75.13)	9.22 (0.97, 87.75)	
Non-Hispanic Black	1.52 (0.11, 21.93)	1.40 (0.10, 19.50)	1.97 (0.15, 26.70)	
Other Race	5.51 (0.30, 99.87)	5.80 (0.34, 98.74)	7.44 (0.44, 126.04)	
Education level				0.1684
Below high school	Ref.	1.17 (0.64, 2.12)	1.74 (0.93, 3.23)	
High school	6.57 (0.64, 67.35)	3.98 (0.39, 40.65)	6.08 (0.60, 61.72)	
Above high school	6.67 (0.84, 52.77)	7.19 (0.91, 56.76)	7.91 (1.01, 62.16)	
Marital Status				0.7462
Cohabitation	Ref.	1.02 (0.78, 1.33)	1.30 (0.96, 1.77)	
Solitude	1.55 (0.27, 8.79)	1.40 (0.25, 7.81)	2.17 (0.40, 11.75)	
Never married	0.38 (0.03, 4.35)	0.37 (0.03, 4.34)	0.35 (0.03, 4.01)	
PIR				0.6787
<1.3	Ref.	1.20 (0.71, 2.03)	1.56 (0.92, 2.64)	
≥ 1.3–<3.5	8.47 (1.58, 45.45)	8.68 (1.61, 46.65)	10.92 (2.05, 58.25)	
≥ 3.5	6.02 (0.89, 40.89)	5.20 (0.78, 34.71)	5.58 (0.81, 38.30)	
BMI (kg/m2)				0.2065
<25	Ref.	0.57 (0.28, 1.15)	1.10 (0.56, 2.17)	
≥ 25–<30	2.61 (0.29, 23.57)	2.03 (0.22, 18.40)	2.89 (0.32, 26.27)	
≥ 30	3.48 (0.46, 26.48)	4.18 (0.55, 31.60)	4.91 (0.65, 37.05)	
Physical activity				
Vigorous				0.8298
Yes	Ref.	0.86 (0.52, 1.44)	1.18 (0.65, 2.16)	
No	4.41 (0.65, 29.77)	4.53 (0.67, 30.50)	5.76 (0.86, 38.62)	
Moderate				0.8970
Yes	Ref.	0.95 (0.68, 1.33)	1.19 (0.79, 1.78)	
No	1.61 (0.39, 6.69)	1.63 (0.39, 6.73)	2.16 (0.53, 8.84)	
Drinking status				0.9924
Yes	Ref.	0.98 (0.75, 1.29)	1.28 (0.94, 1.75)	
No	2.89 (0.69, 12.20)	2.86 (0.69, 11.91)	3.81 (0.92, 15.77)	
Diabetes				0.0506
Yes	Ref.	0.94 (0.62, 1.43)	0.78 (0.47, 1.29)	
No	0.28 (0.04, 1.99)	0.28 (0.04, 1.99)	0.42 (0.06, 2.94)	
Hypertension				0.5837
Yes	Ref.	1.02 (0.76, 1.38)	1.17 (0.84, 1.64)	
No	0.19 (0.04, 0.81)	0.18 (0.04, 0.76)	0.26 (0.06, 1.11)	
HDL (mg/dL)				0.4361
T1 (10.00–44.00)	Ref.	0.99 (0.66, 1.48)	1.07 (0.69, 1.65)	
T2 (45.00–57.00)	1.40 (0.29, 6.85)	1.23 (0.25, 5.99)	1.57 (0.32, 7.59)	
T3 (58.00–187.00)	1.97 (0.38, 10.05)	2.05 (0.40, 10.55)	3.44 (0.68, 17.56)	
TC (mg/dL)				0.5185
T1 (71.00–164.00)	Ref.	0.99 (0.68, 1.45)	1.06 (0.68, 1.63)	
T2 (165.00–199.00)	1.37 (0.30, 6.28)	1.43 (0.31, 6.57)	2.36 (0.52, 10.76)	
T3 (200.00–446.00)	3.64 (0.80, 16.63)	3.51 (0.76, 16.18)	4.55 (1.00, 20.77)	

Subgroups analysis and interaction tests for the associations between serum cotinine and gallstones in NHANES (2017–2020). The model was adjusted for age, gender, race, education level, marital status, PIR, BMI, physical activity, drinking status, diabetes, hypertension, HDL, and TC except for variables examined in the table. Ref. = 1.0. NHANES, US National Health and Nutrition Examination Survey; PIR, Ratio of family income to poverty; BMI, Body Mass Index; HDL, Body Mass Index; TC, Total Cholesterol.

## 4 Discussion

In this cross-sectional analysis involving 5,856 participants from the NHANES database, we identified a positive correlation between serum cotinine levels and the prevalence of gallstones. This association was further substantiated by curve fitting results. Following subgroup analysis and interaction tests, no confounding factor was identified to potentially influence this relationship. We assert that tobacco exposure, as indicated by serum cotinine concentration, warrants attention, emphasizing the importance of minimizing tobacco exposure or quitting smoking early to prevent gallstones. This finding complements prior research on the association between tobacco exposure and gallstones.

Our study represents the first attempt to utilize serum cotinine concentrations to evaluate the relationship between tobacco exposure status and gallstones. Murray et al. (30) conducted a long-term prospective study involving 46,000 oral contraceptive users in the United Kingdom over 19 years, revealing that smokers had a higher likelihood of developing symptomatic gallstones compared to non-smokers. However, this study could not definitively determine whether the occurrence of gallstones was solely attributed to oral contraceptives and did not investigate the male population. Similarly, Stampfer et al. (31) conducted an 8-year prospective study that identified smoking as an independent risk factor for gallstones after adjusting for BMI, weight change, and other risk factors. However, this study also exclusively focused on the female population, leaving the influence of gender on this association unclear. Kato et al. (32) and Sahi et al. (33) conducted prospective studies on men, both observing a positive correlation between smoking and gallstones. However, these studies faced challenges in fully excluding the influence of race and post-immigrant lifestyle, and the latter did not find a statistically significant association between smoking duration and gallstones. Misciagna et al. (34) conducted a long-term follow-up study in a small town in southern Italy, involving 3,500 participants, and found a strong association between smoking and gallstones, although no dose–response relationship was evident. Yamada et al. (35) observed a positive association between smoking and gallstones in a prospective study of a Japanese population. However, limitations such as questionnaire-derived smoking data, lack of timely information, and unavailability of data on cigarette or alcohol intake hindered the possibility of conducting a dose–response analysis. Our study addressed some of the limitations of previous research by utilizing extensive and comprehensive data from the NHANES database. Furthermore, the use of serum cotinine concentration to assess smoking status enabled the quantification of tobacco exposure levels, facilitating a more nuanced exploration of the association between tobacco exposure and gallstones through smooth curve fitting.

The observed impact of serum cotinine on gallstone prevalence in this study may be associated with the FXR-megalin/cubilin signaling pathway. Erran et al. (36) discovered that megalin and cubilin proteins are expressed in gallbladder epithelial cells but not in hepatocytes, and their expression is regulated by bile acids rather than cholesterol. This regulation is mediated by the bile acid nuclear hormone receptor farnesoid X receptor (FXR). The FXR-megalin/cubilin signaling pathway plays a role in cholesterol balance regulation and participates in gallstone formation (37). Notably, nicotine inhibits the expression of FXR and megalin in gallbladder epithelial cells (38). When the gallbladder relies on megalin protein for cholesterol transport, this mechanism becomes weakened, leading to disturbances in cholesterol metabolism within the gallbladder. Consequently, this disruption increases the likelihood of cholesterol stone formation. Another potential mechanism involves the down-regulation of megalin expression, disrupting the balance between upstream and downstream signaling pathway components. This disruption may result in an imbalance in bile acid ratios, preventing cholesterol from maintaining its micellar shape. Consequently, cholesterol precipitates as crystals, ultimately leading to gallstone formation (39, 40).

What we have done is the first study to explore the relationship between tobacco exposure and gallstones using serum cotinine concentrations. The strength of this study lies in its utilization of a large and comprehensive dataset derived from the NHANES database, encompassing numerous covariates identified in previous research. Through meticulous adjustment for these confounding factors, we aimed to attain more reliable research outcomes. Additionally, subgroup analyses and interaction tests were conducted to assess the robustness of the findings across different populations and to evaluate the potential impact of various covariates on the results. However, this study also exhibits certain limitations. Despite the consideration of numerous confounding factors, there may still be some omissions, thereby preventing the complete exclusion of potential confounding effects on the results. Furthermore, due to the nature of cross-sectional studies, we are inherently unable to establish a causal relationship between serum cotinine concentration and gallstones. Thus, further investigation is warranted to elucidate the relationship between tobacco exposure and gallstones.

## 5 Conclusion

In this study, it is noteworthy that when serum cotinine concentration exceeded 0.29 ng/mL, each unit increase was associated with a 29% rise in gallstone prevalence. Additionally, curve fitting analysis revealed a positive association between the two variables. These findings prompt further consideration of tobacco exposure and underscore the health risks associated with tobacco and its harmful constituents when burned. Further investigation is needed to clarify the causal relationship between these variables.

## Data availability statement

Publicly available datasets were analyzed in this study. This data can be found at: https://www.cdc.gov/nchs/nhanes/index.htm.

## Ethics statement

The studies involving humans were approved by National Center for Health Statistics (NCHS) Research Ethics Review Board. The studies were conducted in accordance with the local legislation and institutional requirements. The participants provided their written informed consent to participate in this study.

## Author contributions

XL: Methodology, Writing – original draft, Writing – review & editing, Project administration, Software. ZZ: Data curation, Writing – original draft, Software. HW: Formal analysis, Writing – original draft, Software. SF: Validation, Writing – original draft, Investigation. MH: Project administration, Writing – original draft, Validation. ST: Conceptualization, Writing – original draft, Methodology. YL: Conceptualization, Methodology, Writing – review & editing, Funding acquisition.

## Footnotes

^1^ www.empowerstats.net
